# Drag Reduction Performance and Mechanism of Hydrophobic Polymers in Fresh Water and Brine

**DOI:** 10.3390/polym12040955

**Published:** 2020-04-20

**Authors:** Hongzhong Tan, Jincheng Mao, Wenlong Zhang, Bo Yang, Xiaojiang Yang, Yang Zhang, Chong Lin, Jianfa Feng, Hao Zhang

**Affiliations:** 1 State Key Laboratory of Oil and Gas Reservoir Geology and Exploitation, Southwest Petroleum University, Chengdu 610500, China; thzswpu1992@163.com (H.T.); xjyang@swpu.edu.cn (X.Y.); yangzhang10234@163.com (Y.Z.); 201999010016@swpu.edu.cn (C.L.); 2 Institute of Engineering Technology, The Fourth Oil Extraction Plant of Huabei Oilfield Company, CNPC, Langfang 065000, China; f18603175323@163.com; 3 Research Institute of Oil and Gas Engineering, Jilin Oilfield Company, CNPC, Songyuan 138000, China; 13086655175@163.com

**Keywords:** hydrophobic associating polymer, drag reducer, salt tolerance

## Abstract

Three kinds of drag reducer were synthesized by inverse emulsion polymerization and named PHWAM-1, PHWAM-2, and PHWAM-3. Drag reduction (DR) tests showed that the three drag reducers have different DR characteristics in fresh water and various saline waters because of their different types of hydrophobic monomers. PHWAM-1, without hydrophobic monomers, performs better in fresh water, while PHWAM-2 and PHWAM-3, with hydrophobic monomers, perform better in brine. In addition, PHWAM-3, which has twin-tailed hydrophobic monomers, performs best in high-concentration brine. Measurements of micro-particle size and observations of spatial structure suggest that although the stronger hydrophobic polymer has no DR advantage over a linear polymer in fresh water, the molecular chains form a mutually associative supporting structure that improves the DR performance over that of a linear polymer in high-concentration brine.

## 1. Introduction

Drag reducers are widely used in the field of fluid transportation. The friction resistance of fluids restricts the flow of fluids in pipelines, resulting in a reduced pipeline transportation capacity and increased energy consumption [1]. The main function of drag reducers is to reduce this resistance and improve the efficiency of fluid transportation. Drag reducers are generally high molecular weight polymers (10^5^~10^7^ g/mol). For example, water-soluble polyethylene oxide can reduce the resistance of water in pipelines by 75%, with an amount of only 25 mg/kg, and can increase the rate of the effluent by several times [2], which can be useful in extinguishing fires or other emergency applications. Firefighters in New York used water-soluble polymers to increase the flow of drainage systems in the early 20th century [3].

Drag reducers are extensively used in the petroleum industry. For example, drag reducers are indispensable additives in transnational oil and gas pipeline transportation. During the operation of hydraulic fracturing, where the frictional resistance of fluid is more obvious because the fracturing fluid needs to be delivered into the formation through pipelines several kilometers long, using great pumping pressure [4,5,6], drag reducers are absolutely indispensable to ensure success.

The water quality of the solution the drag reducer is added to has a significant impact on the drag performance. Research has shown that inorganic ions, especially divalent metal ions, greatly influence the performance of drag reducers. The viscosity and drag reduction (DR) efficiency of a solution will be greatly reduced, and flocculation or sedimentation may occur, if Ca^2+^ concentration exceeds 200 mg/L or Mg^2+^ concentration exceeds 100 mg/L [7,8].

In recent years, research and development of salt resistant drag reducers for aqueous solutions have received continuous attention, due to the urgent demands of the market. In 2009, Aften et al. synthesized a copolymer, used as a drag reducer, that can be used in purified water and mineralized water, and the DR is 52% in mineralized water with 2% KCl [9]. In 2014, Zhou et al. developed the drag reducer called FRPW, which can be used to prepare slick water for hydraulic fracturing by using mineralized water with 10^5^ mg/L salt content; the total content of Ca^2+^ and Mg^2+^ was 1500 mg/L [10]. Paul patented a polyacrylamide slick water with good salt tolerance. The polymer is a copolymer of acrylamide and acrylic acid and a copolymer of acrylamide and a cationic monomer of quaternary ammonium salt. The patent claimed that the drag reducer had high DR even at a salinity of 1 × 10^5^ to 1.5 × 10^5^ mg/L [11].

Though current research has made some achievements in salt tolerance, it has mainly been aimed at monovalent salts (Na^+^, K^+^) [12]. Studies on the solubility and DR of drag reducers in brine with a high content of bivalent Ca^2+^ and Mg^2+^ (>1.0 × 10^4^ mg/L) have rarely been reported. The development of a drag reducer with a high tolerance to bivalent cations such as Ca^2+^ and Mg^2+^ is an important research direction to broaden the application of drag reducers.

In this paper, three kinds of polymer drag reducer are designed, named PHWAM-1, PHWAM-2, PHWAM-3, according to the characteristics of salt-resistant. All of them have acrylamide (AM) as the main structure, because AM can be initiated to form highly active radicals and obtain high molecular weight linear polymers. The water solubility of the linear polymer is enhanced by the introduction of acrylic acid (AA). The introduction of 2-acrylamido-2-methylpropane sulfonate (AMPS) enables the molecular chain to carry a large side group of branched chains, which inhibits the curling motion of the molecular chain. PHWAM-1 (AM/AA/AMPS) has no hydrophobic group. In contrast, the polymer chains of PHWAM-2 and PHWAM-3 have different hydrophobic groups in main chains. The hydrophobic groups on the molecular chains can make the polymer chains associate with each other and maintain a certain degree of extension because of the hydrophobic association effect, so that the drag reducers can maintain good DR in a high-concentration brine. By comparing the DR performance of the three drag reducers in water and brine, the DR and salt tolerance mechanisms of the polymers containing hydrophobic groups are analyzed, which provides new insight for the development of drag reducers.

## 2. Material and Methods

### 2.1. Raw Materials

Raw materials of AM, AA, sodium dodecyl sulfate (SDS), Span-80, Tween-60, sodium chloride (NaCl), magnesium chloride hexahydrate (MgCl_2_·6H_2_O), calcium chloride (CaCl_2_), Acetonitrile, Dichloromethane and white oil were bought from chron chemicals Co.; Ltd. (Chengdu, China). Dodecylamine, 1-bromododecane, 2-methylpropenoyl chloride and AMPS were bought from Shanghai Aladdin BioChem Technology Co.; Ltd. (Shanghai, China). Sodium hydrogen sulfite (NaHSO_3_), ammonium persulphate ((NH_4_)_2_S_2_O_8_), and sodium hydroxide (NaOH) were analytical reagent grade and purchased from Shanghai Macklin Biochemical Technology Co.; Ltd. (Shanghai, China). *N*-n-dodecylacrylamide (C_12_AM), and twin-tailed hydrophobic monomer *N,N*-di-n-dodecylacrylamide (DiC_12_AM) were prepared in the lab by referring to the literature [13,14].

### 2.2. Synthesis of Drag Reducer

Inverse emulsion polymerization was used to synthesize the polymer drag reducers. The process included the preparation of an aqueous phase and an oil phase. Certain amounts of AM (28.43 g, 0.4 mol), AA (10.09 g, 0.14 mol), AMPS (6.21 g, 0.03 mol), C_12_AM (0.28 g, 0.001 mol) or DiC_12_AM (0.47 g, 0.001 mol), and SDS (2.88 g, 0.01 mol) were weighed precisely, with a molar ratio of SDS to hydrophobic monomer of 10, and dissolved in deionized water to form a clarified mixed solution. Then, NaOH solution (30 wt.%) was added until the solution reached a pH of 6 to 7, and the mass of whole aqueous solution was 110 g; in this way, the aqueous phase was obtained. A certain amount of white oil (100 g) was added into a flask, and then Span-80 (8.4 g) and Tween-60 (1.6 g) were added to make the oil phase; a steady system was obtained after stirring at high speed (2000 r/min) for 30 min (Table 1).

**Scheme 1:**
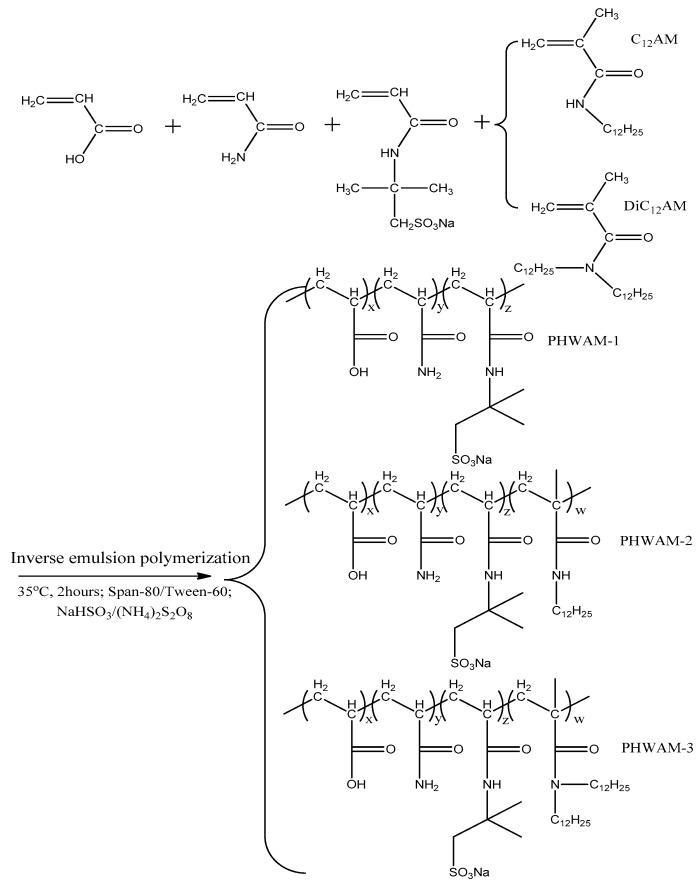
Inverse emulsion polymerization of PHWAM-1/ PHWAM-2/ PHWAM-3.

The water phase was added to the oil phase at a rate of 0.3 mL/s, using a constant pressure dropping funnel, and the system was stirred at high speed (2000 r/min); a white emulsion was obtained. The reaction temperature was 35 °C. Next, 1 wt.% NaHSO_3_ solution (2 mL) and (NH_4_)_2_S_2_O_8_ (1.5 mL) were added to the emulsion, with the whole reaction in a nitrogen atmosphere and stirred at high speed (2000 r/min) for 2 h (Scheme 1).

The drag reducers, each a viscous white emulsion, were obtained after the reaction and named PHWAM-1, PHWAM-2, and PHWAM-3 [15,16].

### 2.3. Methods

#### 2.3.1. Characterization

Each drag reducer emulsion was soaked in anhydrous alcohol for 3 h; the high molecular weight polymer component in the emulsion was insoluble in the anhydrous ethanol solvent, while other components would be completely dissolved. The polymers were precipitated and filtered out, then soaked twice more with anhydrous ethanol, and then precipitated, dried, and cut into small pieces. Finally, pure polymers were acquired after vacuum drying and granulation. Besides, these treated polymers were weighed, and the monomers’ conversion of the inverse emulsion polymerizations was calculated by comparing the dosages of initial monomers. The results showed that the monomers’ conversion of three polymerization reactions were 97.8%, 93.3%, 91.8% respectively.

Nuclear magnetic resonance hydrogen spectra (^1^H NMR) of the polymers were obtained with a Bruker 400 MHz NMR spectrometer (AVANCE III HD 400, Bruker, Karlsruhe, Germany), operating at 400 MHz. Polymers were prepared in D_2_O with a concentration of 10 g/L.

#### 2.3.2. Stability Coefficient

The emulsion of drag reducers was put into scale centrifuge tube and centrifuged for 5 min at 2000r/min in centrifuge (JIDI-4D-WS, Guangzhou, China). The emulsion had a stratification phenomenon after the centrifugation was over, and the stability of the emulsion can be described by the degree of delamination. The method for determining the stability coefficient (R) of emulsion is shown in Formula 1. V_E_ represents the original volume of the emulsion, and V_O_ represents the volume of the separated supernatant oil phase

(1) $$R=\frac{V_{E}-V_{O}}{V_{E}}$$

#### 2.3.3. Dynamic Light Scattering Measurements

The microscopic morphology of the polymers can be indicated by observing their particle size distribution. Dynamic light scattering (DLS) with a wide angle laser light scatterometer (BI-200SM, Brookhaven, Suffolk, NY, USA) is a common method to characterize particle size distribution by measuring the fluctuation of light intensity with time. Solutions of the drag reducers were prepared with different concentrations of brine. The test temperature was 25 °C, the laser module was a 532-Na light source, the detection angle was 90°, and CONTIN software was used for the final analysis of the data.

#### 2.3.4. Microstructure Analysis

The microstructure of the drag-reducer solutions can be observed using a cryo-environmental scanning electron microscope (Cryo-SEM, FEI, Hillsboro, OR, USA), so as to establish the relationship between the microstructure of the polymers and the DR performance, in order to analyze the drag-reducing mechanism of the solutions at different salt concentrations. Solution samples were frozen at −185 °C and then sublimated before observation, keeping the structure of the fluid intact.

#### 2.3.5. DR Testing

**Figure 1:**
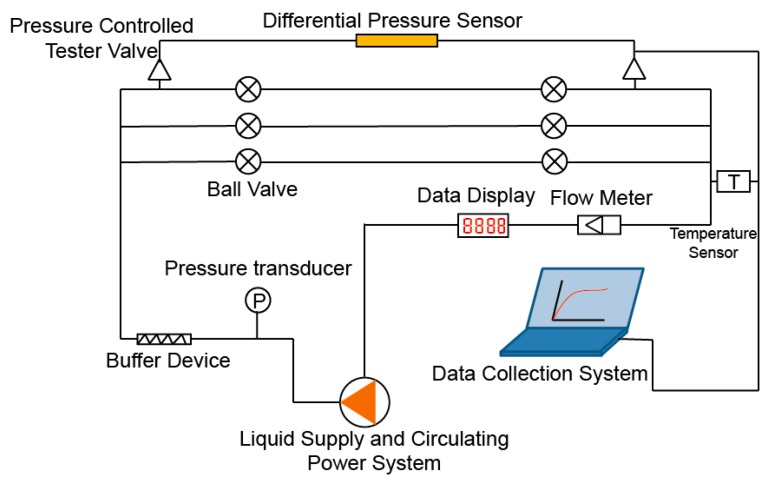
Structural schematic diagram of the MZ-II friction instrument for the drag reduction (DR) test.

Based on analyses of the DR mechanism (turbulence inhibition hypothesis, viscoelastic hypothesis, molecular deformation hypothesis, and so on), combined with the development processes of various kinds of drag reducers (surfactant, polymer, and so on), it has been concluded that pipeline drag can only be reduced by changing the flow shape inside the fluid, inhibiting the fluid turbulence flow inside the pipe, and transferring the activation energy fully into the axial force of the fluid transportation [17,18,19]. A water-soluble linear polymer can inhibit fluid turbulence flow effectively, due to the extensible polymer chains. Long-term research and practice have found that the higher the molecular weight, and the less short-branched chains or more long-branched chains, the better the DR effect. This finding provides an idea for subsequent research and development of drag reducers [20,21,22]. A friction instrument (MZ-II, Hai’an Petroleum Scientific Research Instrument Co.; Ltd.; Hai’an, China) can measure the fluid Reynolds number (*Re*), flow velocity (*u*), and quantity of flow (*Q*) and records the pressure loss ($\Delta P_{f}$) through pressure sensors monitoring the fluid pressure in the pipeline. Fluid density (*ρ*) was set at 1 g/cm^3^. Fluid viscosity (μ) was measured by an Ubbelohde viscometer (0.8 mm diameter). The friction coefficients (λ) of the tested sample liquids were assumed to be the same. The relationship between these parameters is shown in Formulas (2) and (3) [23]. The structure of the instrument is shown in Figure 1; the main components are a liquid supply and circulating power system, data acquisition system, pipeline, and monitoring sensors. The test pipe diameter (*d*) is 4 mm, and the circulating pipeline length (*l*) is 4 m.

(2) $$\Delta P_{f}=\lambda \left(\frac{l}{d}\right)\frac{u^{2}}{2}\rho$$

(3) Re=4Qρ/(πdμ)

The DR calculation is shown in Formula (4) [24]. *∆P* represents the differential pressure produced in the pipeline cycle. *∆P_1_* represents the pressure difference of fresh water. *∆P_2_* represents the pressure difference of the test samples. The paper studied the effect on DR caused by metal ions (Na^+^, Ca^2+^, and Mg^2+^), and the reasons for the variation in the DR performance of the different drag reducers were analyzed

(4) $$DR=\frac{\Delta P1-\Delta P2}{\Delta P1}$$

## 3. Results and Discussion

### 3.1. Characterization

**Figure 2:**
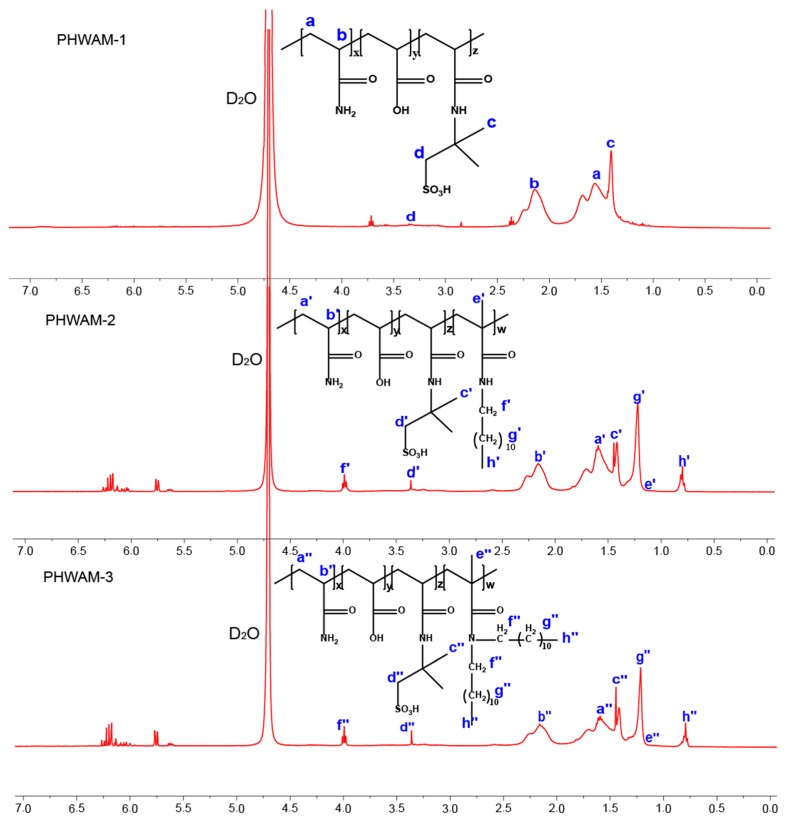
^1^H NMR of drag reducer polymers.

Figure 2 shows the ^1^H NMR (400 MHz, D_2_O) spectrum of the drag reducer polymers. The resonances of the protons are as follows: δ 4.70 (D_2_O) for the solvent peak, δ 1.62 ppm (a, a’, a’’) as the main chain proton peak, δ 2.19~2.31 ppm (b, b’, b’’) for the two protons resonances (–CH_2_–CH–) on the main chain, δ 1.45 ppm (c, c’, c’’) for the methyl proton peaks of the AMPS group segment, δ 3.32 ppm (d, d’, d’’) for the methylene peak attached to the sulfonic acid foundation group of AMPS, δ 3.36 ppm (f’, f’’) for the methylene peak attached to the tertiary amine (C–N), δ 1.23 ppm (g’, g’’) for the methylene overlap peaks of the long chain alkanes, δ 0.80 ppm (h’, h’’) for the methyl proton peak of the long chain alkanes, and δ 1.15 ppm (e’, e’’) for the methyl proton overlap peaks on the main chain. Other peaks are assigned as impurities or solvent, such as δ 3.79 ppm in PHWAM-1 for ethanol solvent, and all the signals above 5 ppm are assigned for unreacted hydrophobic monomers. The results verified that monomers and *N*-n-dodecylacrylamide, *N,N*-di-n-dodecylacrylamide were successfully polymerized by the reverse emulsion polymerization.

### 3.2. Critical Association Concentration

**Figure 3:**
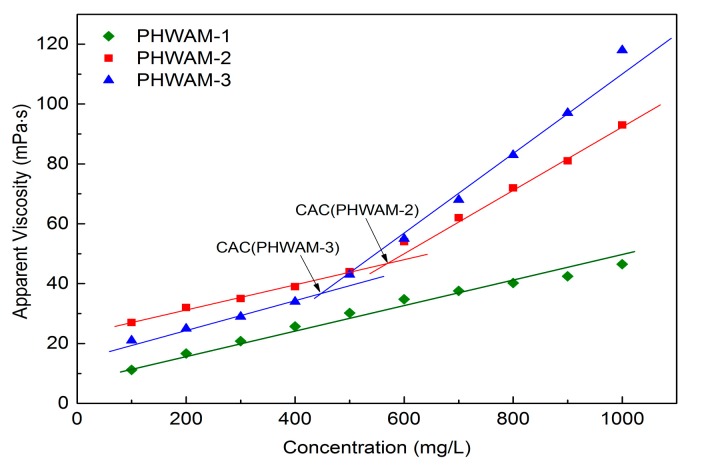
The critical association concentration (CAC) of drag reducer polymers.

Table 2 describes the physical characteristics of three kinds of drag reducer emulsions, including intrinsic viscosity, solid content, and stability coefficient. However, in an aqueous solution, different molecular chains of polymers may have different characteristics. The critical association concentration (CAC) of a polymer can be characterized by the relationship between the concentration and the apparent viscosity of the aqueous solution, as shown in Figure 3 [25]. In the figure, the curves of PHWAM-2 and PHWAM-3 have obvious mutation points, which can stand for the CAC of the polymers. At the CAC, intramolecular association is dominant. Above this concentration, a hydrophobic association between molecules widely occurs, making the solution viscosity increase rapidly. Moreover, the CAC of PHWAM-3 is lower than that of PHWAM-2, which indicates that the hydrophobic unit of PHWAM-3 has a stronger hydrophobic association effect. This also indicates that a double-chain structure has stronger hydrophobicity than a long alkyl chain structure of the same length. The viscosity of the solution prepared with PHWAM-1, without hydrophobic monomers, increases slowly with the increase of concentration, and there is no obvious mutation point. These results show that a long, alkyl-branched chain is necessary for a hydrophobic association.

### 3.3. DR in Fresh Water

**Figure 4:**
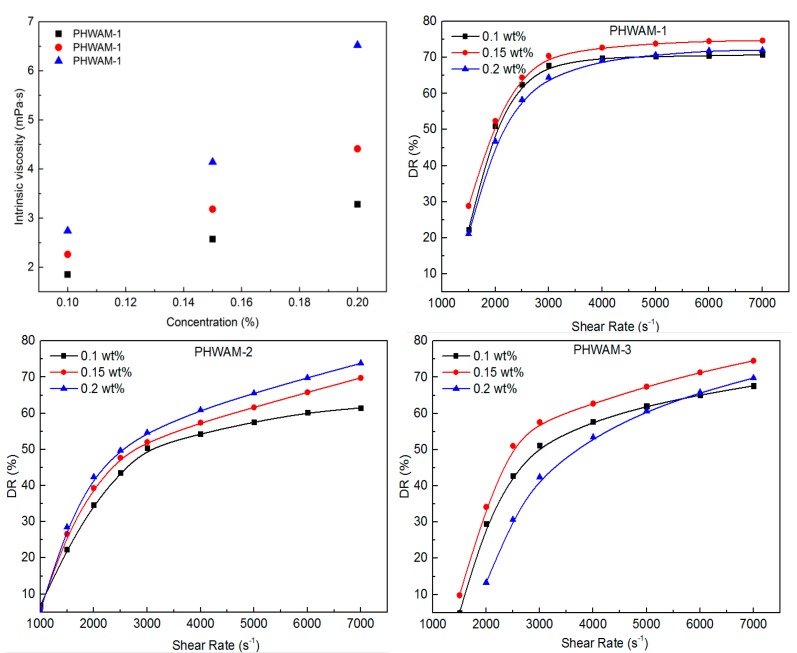
Viscosity and DR properties of drag reducers with different concentrations in fresh water.

In fresh water, the DR performance of each drag reducer was tested by using different concentrations and increasing the shear rate, as shown in Figure 4. The solution viscosity of the drag reducer increases as the concentration grows, but the DR performance of the drag reducers does not increase with the increase in concentration. There is no simple linear relationship between the DR and solution viscosity.

With the increase in drag reducer concentration, the polymer molecules associate with each other, which results in a change of molecular chain morphology. For polymers without a hydrophobic branched chain, the degree of entanglement of the molecular chains increases with increasing concentration [26]. When the shear rate increases gradually, the DR rate first increases sharply and then keeps stable or decreases slightly. When polymer molecular chains are subjected to external shear force, they will change their morphology, thus increasing the DR [27]. When the shear force is insufficient to change the polymer morphology or the degree of change is limited, the DR rate increases more slowly.

**Figure 5:**
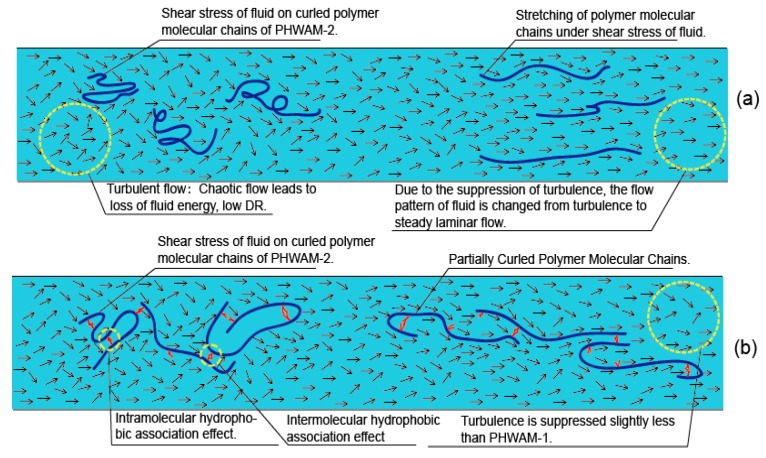
DR mechanism of polymer drag reducers (**a**) without a hydrophobic monomer and (**b**) with a hydrophobic monomer in fresh water.

For linear polymer chains such as PHWAM-1, without hydrophobic groups, slight shear forces can stretch the molecules, so the DR curves increase rapidly. According to the turbulence suppression mechanism hypothesis, extended polymer chains can effectively and rapidly suppress the generation and development of turbulent vortices [28]. When the shear rate reaches 3000 s^−1^, the linear structure of PHWAM-1 has been fully extended, and the turbulent energy can no longer be fully absorbed, so the DR efficiency in the long run is not significantly increased, as shown in Figure 5a.

For PHWAM-2 and PHWAM-3, which contain hydrophobic groups, the intramolecular and intermolecular stretching is not complete. The association structure makes the molecular chains extend to a certain extent but not fully at the initial low shear rate, which is why the DRs of PHWAM-2 and PHWAM-3 are lower than that of PHWAM-1 during the stage in which the DR drastically increases. As the shear force continues to increase, the associating structure can still absorb part of the turbulent energy to make the DR increase slowly, as shown in Figure 5b.

With the increase of concentration, the DR of PHWAM-1 essentially varies little; PHWAM-1 shows the best DR performance at 0.15 wt.% concentration, while the DR decreases slightly when the concentration increases to 0.2 wt.%. According to Virk theory, DR performance has a limit. The greater the concentration of drag reducers, the thicker the elastic buffer layer. When the elastic buffer layer expands to the tube axis, the DR of the drag reducers reaches the limit [29]. Increasing the viscosity of the solution, as a result of increasing the concentration of drag reducer, will increase the load on the pump, thus reducing the fluid transport efficiency.

**Figure 6:**
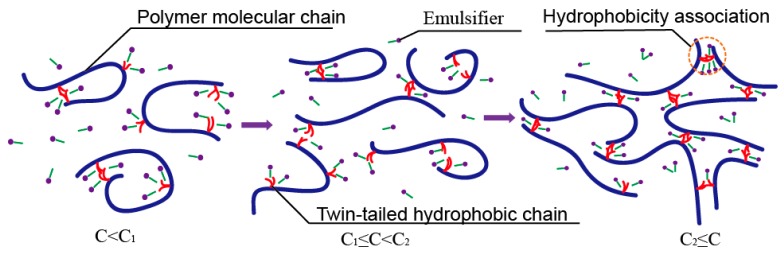
Molecular chain morphology of polymers containing hydrophobic monomers in aqueous solution varies with concentration.

However, the DR performance of PHWAM-2 increases gradually at the same shear rate with the increase of concentration. The polymer content is about 600 mg/L (CAC = 590 mg/L) in 0.2 wt.% PHWAM-2 solution, and the molecular chains of the polymer gradually change from intramolecular association (c < c_1_, c_1_ = initial intermolecular association concentration) to partial intermolecular association (c_1_ < c < c_2_), to large-scale intermolecular association (c > c_2_, c_2_ = CAC). PHWAM-3, which has a smaller CAC than PHWAM-2 and obviously satisfies the above relationship. The stretching degree of the molecular chains increases and the DR performance improves, as shown in Figure 6.

**Figure 7:**
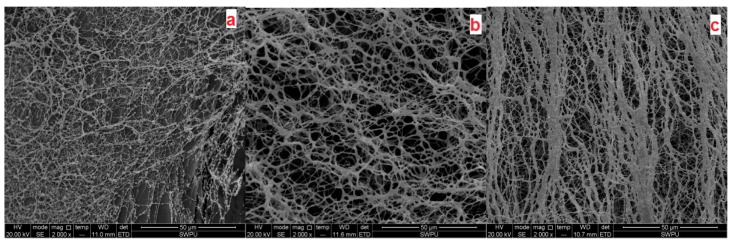
SEM of the polymer molecular chains of the drag reducers in aqueous solutions at different concentrations: (**a**) 0.1 wt.%, (**b**) 0.15 wt.%, and (**c**) 0.2 wt.%.

The DR performance of PHWAM-3 decreases because the polymer content is higher than the CAC (430 mg/L) in 0.2 wt.% PHWAM-3 solution. The solution viscosity increases rapidly when the concentration exceeds 0.15 wt.% and the elastic buffer layer has expanded to the whole diameter of the pipe. The degree of intermolecular entanglement and association of polymers increases sharply when the concentration continues to increase, as shown in Figure 7. In high-speed flowing aqueous solutions, a large amount of energy originally used to transport fluids is absorbed to deform the polymer networks, resulting in a slight reduction in DR efficiency.

In conclusion, in fresh water, the DR performance of the polymers PHWAM-2 and PHWAM-3, with their long alkyl hydrophobic structures, is lower than that of PHWAM-1, but at a high shear rate, the performances are similar. Moreover, the DR performance will be affected when the concentration exceeds the CAC.

### 3.4. DR in Brine

**Figure 8:**
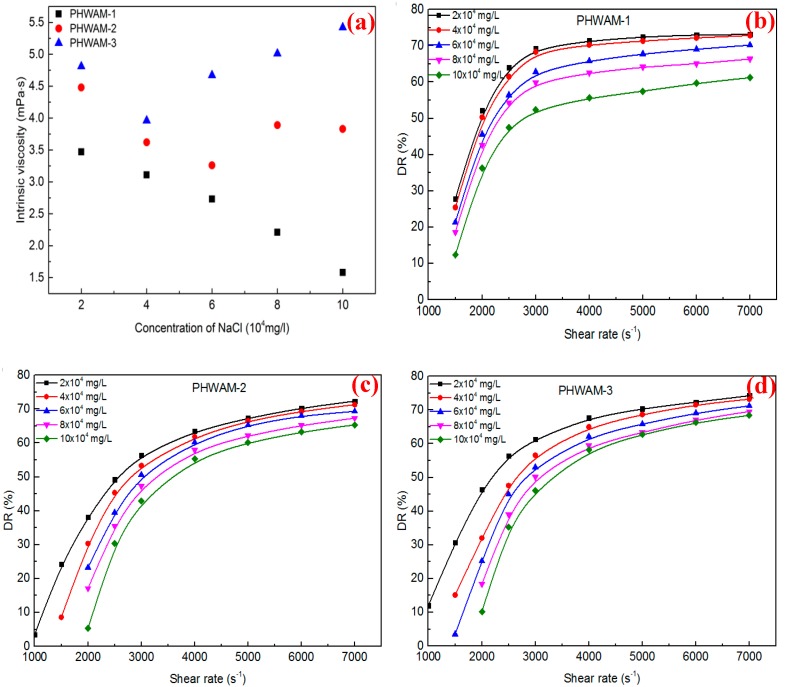
Viscosity(**a**) and DR properties ((**b**) PHWAM-1, (**c**) PHWAM-2, (**d**) PHWAM-3) of 0.15 wt.% drag reducers in NaCl aqueous solutions with different concentrations.

Five kinds of high concentration brine were prepared with NaCl, and the DR effects of the three drag reducers (0.15 wt.%) under different water quality conditions were tested, as shown in Figure 8. The DR performance of the three drag reducers decreased with an increase in NaCl concentration, but the rates of the decline differed. PHWAM-1 decreased the fastest; it decreased from 73.14% DR to 61.24% DR with 2 × 10^4^ mg/L to 10 × 10^4^ mg/L NaCl concentration (5000 s^−1^ shear rate). Under the same conditions, PHWAM-2 decreased from 72.36% DR to 65.38% DR, and PHWAM-3 from 74.25% DR to 68.44% DR.

The data shows that the hydrophobic association groups play a role in salt tolerance, and the stronger the hydrophobicity, the better the salt tolerance effect [30]. Observing changes in the intrinsic viscosity of the drag reducer solutions containing hydrophobic groups (PHWAM-2 and PHWAM-3) shows that the intrinsic viscosity increases at some salt concentrations but not others (Figure 8a); that is, the viscous thickening phenomenon, which is caused by the electrostatic shielding and the compression of the polymer hydration shell, occurs [31,32].

**Figure 9:**
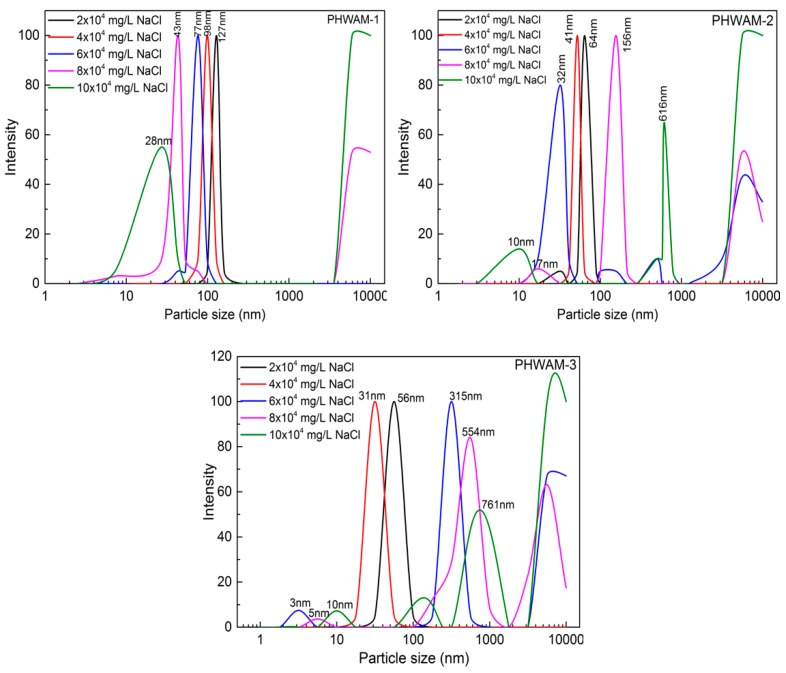
Effect of salt on the particle size distribution of the polymers.

**Figure 10:**
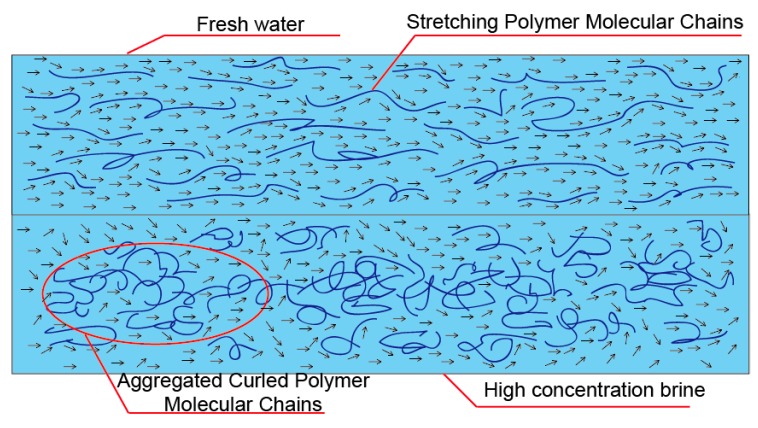
Schematic diagram of the difference in the morphological characteristics of the polymer molecular chains of the drag reducers in fresh water and brine.

DLS was used to analyze the particle size changes of the polymers at different NaCl concentrations, as shown in Figure 9. The particle size distribution of PHWAM-1 decreased with the increase of salt concentration, which indicated that molecular chains of the linear polymer were gradually curled; this result corresponded to the characteristics of decreasing intrinsic viscosity and DR performance. Comparing the spatial structure of PHWAM-1 in fresh water and high concentration brine (10 × 10^4^ mg/L NaCl) shows that PHWAM-1 completely lost its reticulate structure characteristics in brine, indicating that the polymer molecular chains greatly curled, which also explains the drastically reduced DR (Figure 10).

**Figure 11:**
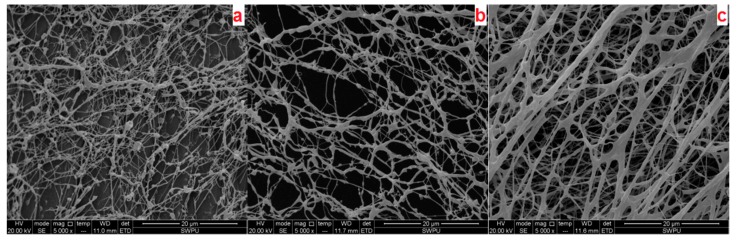
SEM of 0.15 wt.% drag reducers in fresh water: (**a**) PHWAM-1, (**b**) PHWAM-2, and (**c**) PHWAM-3.

However, the intrinsic viscosity and particle size distribution of PHWAM-2 and PHWAM-3 first decreased and then increased. But the DR performance still decreased gradually, which indicates that the increase of intrinsic viscosity and particle size distribution were not caused by the stretching of the polymer molecular chains, due to the increase of salt concentration. In brine, the polarity of the aqueous solution increased, and polymers with hydrophobic groups associated more closely because of the enhanced hydrophobic association effect [33]. The polymer content concentration of 0.15 wt.% PHWAM-2 was lower than that of its CAC, while that of 0.15 wt.% PHWAM-3 was higher than that of its CAC, and the hydrophobic association effect of a single-tail hydrophobic group was lower than that of a double-tail hydrophobic group. Therefore, 0.15 wt.% PHWAM-2 in aqueous solution showed a partially associated, imperfect reticular spatial structure (Figure 11b), while 0.15 wt.% PHWAM-3 in aqueous solution had a more perfect associative reticular spatial structure (Figure 11c).

**Figure 12:**
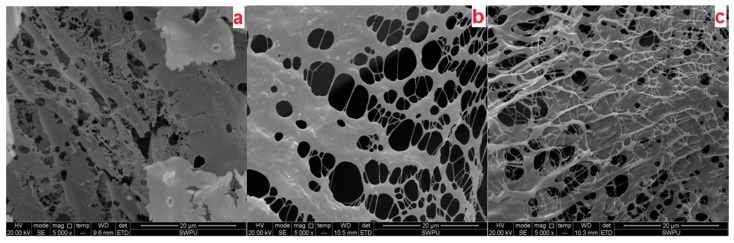
SEM of 0.15 wt.% drag reducers in 10 × 10^4^ mg/L aqueous NaCl solution: (**a**) PHWAM-1, (**b**) PHWAM-2, and (**c**) PHWAM-3.

With the increase in salt content in the aqueous solution, the PHWAM-2 polymer molecule chains became more curly, due to association; that is, the hydraulic radius decreased, and the particle size distribution decreased gradually, so the DR performance decreased. The hydrophobic association effect continued to be enhanced, with the increase of salt concentration leading the intermolecular association to appear on a large scale, even a supramolecular network structure of aggregates, which made the particle size distribution increase [34]. However, the three-dimensional reticular spatial structure was different from that of an aqueous solution without inorganic salts, as shown in Figure 12. In 10 × 10^4^ mg/L aqueous NaCl solution, the polymer reticular spatial structure was relaxed and easily destroyed at a high shear rate (Figure 12a,b), so the polymer’s DR performance was low. For PHWAM-3 with its stronger hydrophobicity, the strength of the spatial reticulate structure was stronger than that of PHWAM-2 because the initial network structure had been formed. In 10 × 10^4^ mg/L aqueous NaCl solution, the polymer reticulate structure of PHWAM-3 was still relatively perfect and the skeleton structure was clear (Figure 12c), which shows that the solution had a better high-speed shear resistance and could maintain a better DR performance.

The morphology of the polymer molecular chains is the ultimate manifestation of the interaction for electrostatic shielding effect, hydrophobic association effect, compressive hydration shell effect. Although the hydrophobic association effect is enhanced by the addition of inorganic salts, the effect of electrostatic shielding and the double layer compression of the hydration shell on the molecular chains is greater, which makes the molecular chains curl again, and reduces the particle size distribution and DR performance [35]. When higher salt content makes the association effect stronger than other factors, the intermolecular association effect forms and the particle size distribution of the molecular chains and the intrinsic viscosity both increase.

**Figure 13:**
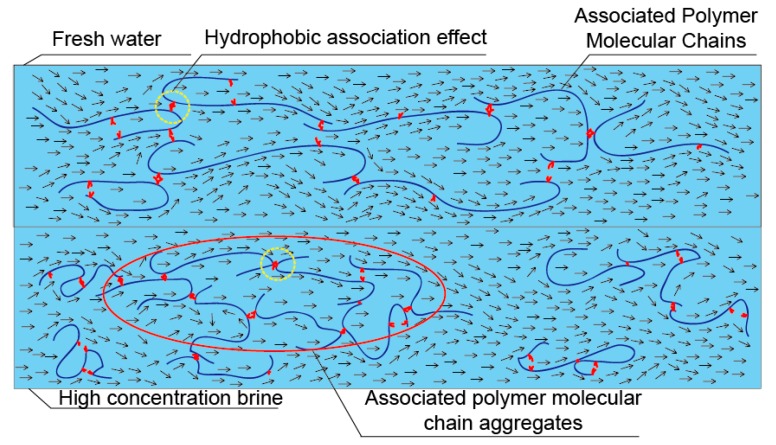
DR effect of polymer molecular chains containing hydrophobic monomers in fresh water and brine.

On the one hand, the elasticity of polymer molecular chains is greatly restricted by the entanglement and association of the chains. This restricted elasticity makes it impossible to absorb the turbulent energy effectively and release it into the central layer of the fluid [36]. On the other hand, a high concentration of inorganic salts can still crimp the molecular chain segments that lack hydrophobic groups, AMPS groups, and large side groups [37]. Therefore, DR performance will still be reduced. However, due to the hydrophobic association effect and the mutual association of molecular chains, the molecular chains still have a stretching structure (Figure 13). In brine, the DR performance of PHWAM-3 was better than that of PHWAM-1, and the hydrophobicity of the double tail was stronger, making the DR performance slightly better than that of PHWAM-2.

### 3.5. DR of High Mineralization Water Quality

**Figure 14:**
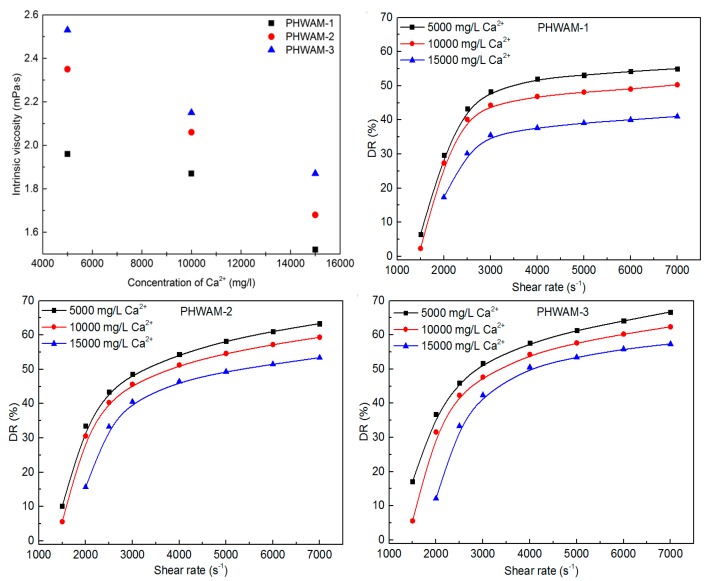
Viscosity and DR performance of 0.15 wt.% drag reducers in brine (10 × 10^4^ mg/L) with different Ca^2+^ concentrations: 5000 mg/L, 10,000 mg/L, and 15,000 mg/L.

Different inorganic salt ions have different effects on the polymer. Because the charge of a divalent cation is twice that of a monovalent cation, the effect of Ca^2+^ and Mg^2+^ ions on polymer solution is much stronger than that of Na^+^ or K^+^ [38]. In fact, Ca^2+^ and Mg^2+^ can bridge polymer molecular chains containing COO^−^ groups [39]. Viscosity and DR performance of the three drag reducers in high salinity water containing Ca^2+^ are shown in Figure 14.

The DR performance decreased in water containing different Ca^2+^ concentrations (5000 mg/L, 10,000 mg/L, and 15,000 mg/L) with a total salinity of 10 × 10^4^ mg/L: PHWAM-1 decreased from an initial DR of 61.24% (0 mg/L Ca^2+^) to 41.12% DR (15,000 mg/L Ca^2+^); that of PHWAM-2 decreased from 64.38% DR to 53.41% DR; and that of PHWAM-3 decreased from 68.44% DR to 57.35% DR (Table 3). The analysis of the DR variation law shows that the effect of Ca^2+^ on drag reducers is more significant than that of Na^+^, because a small content of Ca^2+^ can reduce the DR performance.

Comparing the DR of the drag reducers shows that the drag reducers PHWAM-2 and PHWAM-3, containing hydrophobic groups, had a better DR performance in the presence of Ca^2+^. The DR decrease was very small (1.05% and 1.71%, respectively), while the DR decrease of PHWAM-1 was 6.21% at 5000 mg/L Ca^2+^ concentration. When the Ca^2+^ concentration reached 15,000 mg/L, PHWAM-3 maintained the highest DR performance, and PHWAM-2 also had a higher DR performance than PHWAM-1. These results indicate that a polymer drag reducer with a hydrophobic, long-chain structure can maintain better DR performance in a high mineral brine, and the stronger the hydrophobic association property, the higher the DR performance that can be maintained.

**Figure 15:**
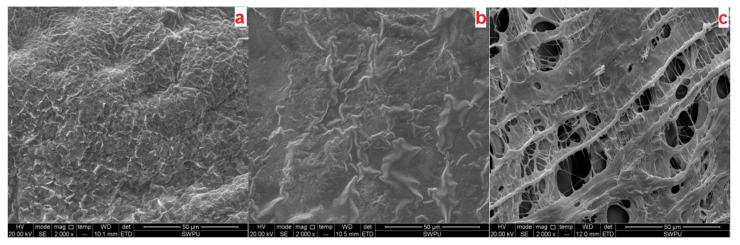
SEM of 0.15 wt.% drag reducers in brine (10 × 10^4^ mg/L) containing Ca^2+^ at 10,000 mg/L: (**a**) PHWAM-1, (**b**) PHWAM-2, (**c**) PHWAM-3.

Observing the micro-morphology of the three drag reducers in brine (10 × 10^4^ mg/L) containing high concentration of calcium ions (10,000 mg/L) shows that PHWAM-1 and PHWAM-2 had no clear spatial network structure, while PHWAM-3 still had a local network structure (Figure 15), which demonstrates that a polymer with stronger hydrophobicity is more tolerant to Ca^2+^, so that it better maintains its original structural characteristics, thus maintaining a better DR performance.

**Figure 16:**
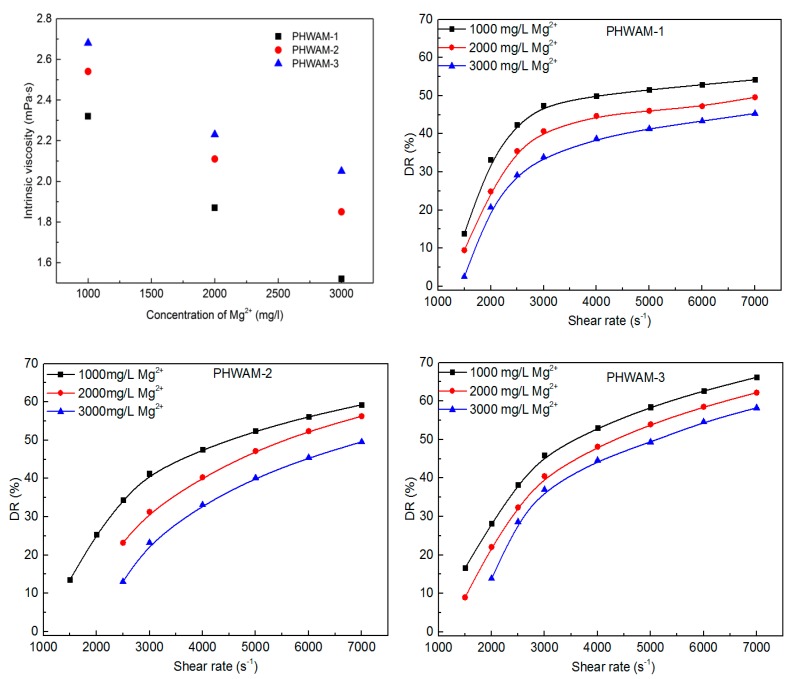
Viscosity and DR properties of 0.15 wt.% drag reducers in brine (10 × 10^4^ mg/L) with different Mg^2+^ concentrations: 1000 mg/L, 2000 mg/L, and 3000 mg/L.

In water with the same total salinity, the DR performance of drag reducers at different Mg^2+^ contents (1000 mg/L, 2000 mg/L, and 3000 mg/L) was tested, as shown in Figure 16. The DR performance of PHWAM-1 decreased from an initial DR of 54.18% (1000 mg/L Mg^2+^) to 45.32% DR (3000 mg/L Mg^2+^); that of PHWAM-2 decreased from 59.27% DR to 49.58% DR; and that of PHWAM-3 decreased from 66.18% DR to 58.24% DR (Table 4). Comparing with the test results of the drag reducers in the water containing Ca^2+^ shows that Mg^2+^ has a greater influence on polymer drag reducers than does Ca^2+^. The 3000 mg/L Mg^2+^ can greatly reduce DR performance, as much as the 15,000 mg/L Ca^2+^. Although Ca^2+^ and Mg^2+^ have the same charge, the ionic radius of Mg^2+^ is smaller, and the interaction with water molecules is stronger; therefore, the ability to compress the polymer hydration shell and curl the polymer molecular chains is stronger.

**Figure 17:**
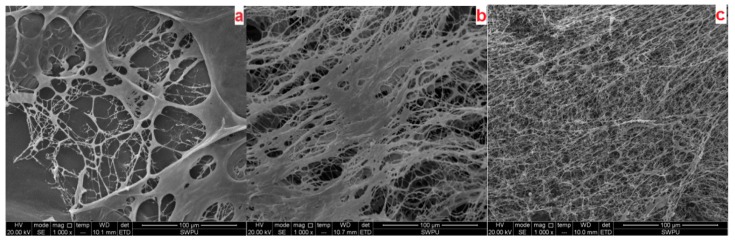
SEM of 0.15 wt.% drag reducers in brine (10 × 10^4^ mg/L) containing Mg^2+^ (1000 mg/L): (**a**) PHWAM-1, (**b**) PHWAM-2, and (**c**) PHWAM-3.

Comparing the effects of the drag reducers shows that the PHWAM-3, containing double-tailed, long alkyl hydrophobic groups, had a higher DR retention rate than PHWAM-2 or PHWAM-1. In addition, the DR effect of PHWAM-2 was better than that of PHWAM-1. Comparing the structural network integrity of the three drag reducers in aqueous solutions containing Mg^2+^ shows that PHWAM-3 could maintain a very high degree of spatial network structure (Figure 17), thus retaining a better DR performance.

These results indicate that using a hydrophobically associative polymer as a drag reducer can produce a better DR performance in brine with a high concentration of Ca^2+^ and Mg^2+^.

## 4. Conclusions

To study the salt resistance of drag reducers, three kinds of polymer drag reducer (named PHWAM-1, PHWAM-2, and PHWAM-3) were designed and synthesized. The structures of PHWAM-2 and PHWAM-3 contain special hydrophobic associating groups (C_12_AM and DiC_12_AM, respectively). The mechanism of the drag reducers in brine was analyzed through testing in fresh water and various brine solutions, using particle size measurement and microstructure observation of the drag reducers. Based on the experiments in this paper, the following conclusions can be drawn.

(1) PHWAM-1 can achieve a high DR performance in fresh water, due to its more-stretched molecular chain under a low *Re*. PHWAM-2 and PHWAM-3 showed potential DR properties only under the action of high-speed fluid shear stress, due to the intramolecular hydrophobic association effect.

(2) In fresh water, when the concentration of polymer in a drag reducer exceeds its CAC, the DR performance will decrease, due to the reduced extension of the molecular chains because of intermolecular association.

(3) In brine, the hydrophobic association effect can effectively maintain a certain degree of molecular chain extension, thus maintaining a better DR effect, and the stronger the hydrophobic association effect, the more obvious the DR effect.

(4) The effect of divalent ions on DR performance is much greater than that of monovalent ions, and the effect of Mg^2+^ is greater than that of Ca^2+^.

## Figures and Tables

**Table 1:** The synthetic scheme of drag reducers.

Samples	Water Phase (110 g, pH = 7)						Oil Phase (110 g, HLB = 6)		
	AM (mol)	AA (mol)	AMPS (mol)	C^12^AM (mol)	DiC^12^AM (mol)	SDS (mol)	White Oil (g)	Tween-60 (g)	Span-80 (g)
**PHWAM-1**	0.4	0.14	0.03	\	\	0.01	100	1.6	8.4
**PHWAM-2**	0.4	0.14	0.03	0.001	\	0.01	100	1.6	8.4
**PHWAM-3**	0.4	0.14	0.03	\	0.001	0.01	100	1.6	8.4

**Table 2:** The characteristics of the drag reducers samples.

Samples	Intrinsic Viscosity (mPa·s)	Solid Content (%)	Stability Coefficient
PHWAM-1	16.6	20.0	0.91
PHWAM-2	15.6	19.1	0.87
PHWAM-3	15.4	18.9	0.84

**Table 3:** The effect of Ca^2+^ on samples in brine (10 × 10^4^ mg/L).

Sample of Reducers	DR of Reducers		
	0 mg/L Ca^2+^	5000 mg/L Ca^2+^	15,000 mg/L Ca^2+^
PHWAM-1	61.24%	55.03%	41.12%
PHWAM-2	64.38%	63.33%	53.41%
PHWAM-3	68.44%	66.73%	57.35%

**Table 4:** The effect of Mg^2+^ on samples in brine (10 × 10^4^ mg/L).

Sample of Reducers	DR of Reducers		
	1000 mg/L Ca^2+^	2000 mg/L Ca^2+^	3000 mg/L Ca^2+^
PHWAM-1	54.18%	48.77%	45.32%
PHWAM-2	59.27%	57.12%	49.58%
PHWAM-3	66.18%	62.51%	58.24%
